# The Dilemma of Analyzing Physical Activity and Sedentary Behavior with Wrist Accelerometer Data: Challenges and Opportunities

**DOI:** 10.3390/jcm10245951

**Published:** 2021-12-18

**Authors:** Zan Gao, Wenxi Liu, Daniel J. McDonough, Nan Zeng, Jung Eun Lee

**Affiliations:** 1 School of Kinesiology, University of Minnesota—Twin Cities, 1900 University Ave. SE, Minneapolis, MN 55455, USA; 2 Department of Physical Education, Shanghai Jiao Tong University, Shanghai 200240, China; liux4443@umn.edu; 3 Division of Epidemiology and Community Health, School of Public Health, University of Minnesota—Twin Cities, 420 Delaware St. SE, Minneapolis, MN 55455, USA; mcdo0785@umn.edu; 4 Prevention Research Center, Department of Pediatrics, University of New Mexico Health Sciences Center, Albuquerque, NM 87131, USA; NZeng@salud.unm.edu; 5 Department of Applied Human Sciences, University of Minnesota—Duluth, 1216 Ordean Court SpHC 109, Duluth, MN 55812, USA; junelee@d.umn.edu

**Keywords:** cut points, deep learning, GGIR, machine learning, motion sensors, steps per minute

## Abstract

Physical behaviors (e.g., physical activity and sedentary behavior) have been the focus among many researchers in the biomedical and behavioral science fields. The recent shift from hip- to wrist-worn accelerometers in these fields has signaled the need to develop novel approaches to process raw acceleration data of physical activity and sedentary behavior. However, there is currently no consensus regarding the best practices for analyzing wrist-worn accelerometer data to accurately predict individuals’ energy expenditure and the times spent in different intensities of free-living physical activity and sedentary behavior. To this end, accurately analyzing and interpreting wrist-worn accelerometer data has become a major challenge facing many clinicians and researchers. In response, this paper attempts to review different methodologies for analyzing wrist-worn accelerometer data and offer cutting edge, yet appropriate analysis plans for wrist-worn accelerometer data in the assessment of physical behavior. In this paper, we first discuss the fundamentals of wrist-worn accelerometer data, followed by various methods of processing these data (e.g., cut points, steps per minute, machine learning), and then we discuss the opportunities, challenges, and directions for future studies in this area of inquiry. This is the most comprehensive review paper to date regarding the analysis and interpretation of free-living physical activity data derived from wrist-worn accelerometers, aiming to help establish a blueprint for processing wrist-derived accelerometer data.

## 1. Introduction

Physical behaviors (i.e., physical activity (PA) and sedentary behavior) have been the focus among many researchers in the biomedical and behavioral science fields. Individuals’ physical behaviors play critical roles in their health outcomes and thus, studying these behaviors has become highly prevalent over the past decade [1,2]. In addition to behavioral change interventions, accurately measuring these behaviors is important for monitoring surveillance and implementing interventions within the scientific community. As is known, since the 1980s, physical behaviors have been quantified through self-reported questionnaires and diaries, which are generally limited in their accuracy due to recall bias and measurement errors. With recent advancements in technology, measurement instruments are rapidly evolving. Among them, wearable motion sensors, such as accelerometers, have gained momentum since the 1990s [3]. Specifically, accelerometers refer to device-based motion sensors that capture changes in individuals’ gravitational accelerations and provide detailed movement information, such as movement directions and duration. More recently, accelerometers have been widely accepted as the preferred device-based instrument for assessing PA and sedentary behaviors in laboratory- and population-based settings due to their accuracy, cost-effectiveness, and unobtrusive nature (i.e., low participant burden) [4].

Accelerometers of earlier generations simply performed onboard signal processing and hence only stored derived output with the goal of reducing battery consumption and minimizing memory requirements. Yet, with technological evolution and the advocacy of the movement towards more open and transparent science, accelerometers have become smaller, cheaper, and more power efficient, and can store raw data for offline processing and analysis. The raw data are typically expressed in gravitational accelerations in different dimensions, thereby providing both movement and gravitational data [5]. On the other side, this switch is counterbalanced by the large magnitude of data collected during one single measurement period. For example, a one-week measurement period may result in several thousand raw data points. This calls for the need to process the raw data to generate meaningful outcomes for use in scientific communities. Currently, there are several raw accelerometer data processing methods to objectively analyze physical behaviors [3,6]. Yet, there is little consensus regarding the most appropriate methods to generate meaningful and interpretable activity metrics (e.g., cut point-free accelerometer metrics) that can be used to facilitate comparison and data harmonization across studies [7].

Recently, there has been a shift from the traditional, hip-worn monitor placement of accelerometers to wrist-worn placement [8,9]. However, data analysis strategies and methods developed for hip-worn accelerometers do not practically translate to that of wrist-worn accelerometers. In fact, the widely used cut points used for quantifying activity yield several major concerns, as follows: (1) cut points are protocol- and population-specific (e.g., age group), leading to results that are not comparable across studies; (2) two participants with similar levels of activity score very differently if one has activity falling just above the cut point and one has activity falling just below the cut point; (3) many participants fail to obtain any activity above cut points—particularly in the vigorous intensity range—and consequently, a large number of participants simply score zero active minutes. There is a consensus nowadays that, when using cut points, we will never truly know the actual PA levels and the prevalence of meeting the PA guidelines among healthy and clinical populations based on accelerometer data. In particular, a number of empirical studies and reviews have identified currently available evidence concerning the use of accelerometers for PA measurement among various clinical populations, including patients with diabetes [10], knee osteoarthritis [11], chronic health conditions [12], and overweight and obesity [13]. To this end, accurately analyzing and interpreting wrist-worn accelerometer data has become a major challenge facing many healthcare clinicians and researchers. In response, this paper attempts to review different methods for analyzing wrist-worn accelerometer data and offers cutting edge, yet appropriate analysis plans for analyzing these data in the assessment of physical behavior. Our goal is to help establish a blueprint for processing wrist-derived accelerometer data for clinicians and researchers alike. Accordingly, in this paper, we first provide the fundamentals of wrist-worn accelerometer data, followed by various methods of dealing with these data (e.g., GGIR, machine learning) and discuss opportunities, challenges, and directions for future studies in this area of inquiry.

## 2. Fundamentals of Accelerometer-Derived Data

Given the scope of this methodology paper, we will only describe device type and device placement in this section. Details concerning accelerometer sampling frequency, filter, epoch length, wear time, non-wear time, valid wear day, and valid wear week can be found in the literature [14,15].

### 2.1. Device Type

Accelerometers assess the acceleration of the body in one or more planes of movement. They are small, portable motion sensors that record second-by-second information about the frequency, duration, intensity, and patterns of one’s body movement. That is, accelerometers are designed to assess total ambulatory activity levels via the measurement of accelerations and are therefore capable of providing an estimate of energy expenditure [16,17]. Generally, there are uniaxial and triaxial accelerometers. The uniaxial accelerometer measures acceleration in a single plane (usually the vertical plane), such as the Actical accelerometer. Triaxial accelerometers, on the other hand, measure acceleration in the vertical, horizontal, and mediolateral planes, such as the GENEActiv and ActiGraph accelerometers. For example, the ActiGraph accelerometer (recent models: GT3X+, Link GT9X) is the most popular motion sensor for activity research these days, as this accelerometer can provide very detailed information regarding one’s PA intensity (sedentary, light, moderate, and vigorous), time spent at different PA intensities, estimations of energy expenditure, steps, and raw counts, among others. Accelerometers have shown acceptable validity and reliability and have been acknowledged as accurate objective instruments for PA measurement among various populations [17] in surveillance studies and intervention studies across the world.

### 2.2. Device Placement

In general, accelerometers can be worn on various locations of the body (e.g., hip, wrist, thigh, ankle, chest). Among them, the hip is the most common placement site, particularly for large epidemiological and intervention studies [6,18]. Yet, in recent years, there has been a shift from placement at the hip to placement at the wrist. For example, the National Health and Nutrition Examination Survey (NHANES) previously employed hip-worn uniaxial accelerometers to measure PA (2003–2004 and 2005–2006) but this has recently changed to wrist-worn triaxial accelerometers during recent population-level PA surveillance (2011–2014) among individuals aged ≥6 years [6]. Some advantages of the wrist-worn placement include improved user compliance (e.g., increased wear compliance, longer wear times), being perceived as less intrusive, with an improved capability of assessing sleep behaviors, and with the capability of capturing more upper extremity movements which cannot be captured with hip-placed devices [19,20,21,22]. For example, in Fairclough et al.’s study [22], the compliance rate for wrist wearing was 89%, as compared with 19% waist wearing among children aged 9–10 years.

The NHANES placed accelerometers on individuals’ non-dominant wrist [7,23,24]. The underlying rationale of such a recommendation is that placement of the monitor on the dominant wrist may misclassify time spent in sedentary behavior as time spent in PA [14]. In other words, it is possible that dominant wrist placement may increase an individual’s activity counts during sedentary behaviors that involve upper extremity movement, such as drawing, coloring, writing, and playing with mobile electronic devices, resulting in continued accumulation of PA while the individual is actually sedentary. On the contrary, if the accelerometer is placed on the non-dominant wrist, there should be limited miscalculated activity counts. Therefore, to accurately distinguish time in sedentary behavior and light-intensity PA—the activity intensity adjacent to sedentary behavior—researchers recommend that wrist-worn accelerometers be placed on the non-dominant wrist while assessing physical behaviors [25,26]. The similarities and differences of wrist- and hip-worn accelerometers are shown in Table 1.

## 3. Approaches in Processing Wrist Accelerometer Data

As stated earlier, with the advancement of technology, accelerometers are now being widely used in many settings and among various populations for objectively assessing physical behaviors for a typical 24 h/day, 7 days/week. Notably, only the accelerometer placement and sampling frequency criteria have to be determined prior to the assessments, whereas the rest of data processing options can be decided upon later. Although the currently available accelerometers are able to assess physical behaviors, the company-specific, proprietary algorithms make it difficult to compare the data from different studies using different accelerometers. Thus, the agreement of outputs that are generated from different accelerometers remain questioned across studies. In the following section, we will introduce two major approaches (cut point- or threshold-based approaches and machine learning approaches) that have been used to process wrist accelerometer data. Cut point- or threshold-based approaches are widely used as an approach in processing accelerometer data into physical behavior outcomes, using either activity counts or gravitational units. Machine learning, on the other hand, is another approach in generating predictive models with the goal of converting raw accelerometer signal (data) into physical behavior outcomes.

### 3.1. Cut Point- or Threshold-Based Approaches

#### 3.1.1. Activity Count-Based Cut Points

**Figure 1:**
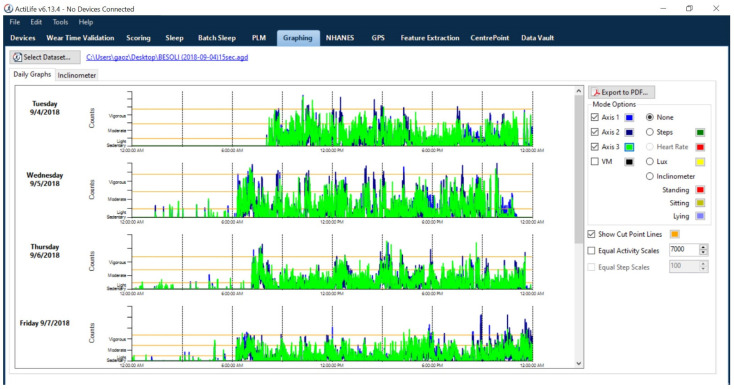
Visual outputs of daily PA from ActiLife v6.13.4 (ActiGraph software).

To sort and analyze accelerometer data, researchers have developed various cut points or regression equations using vector magnitude (i.e., the square root of the sum of squares of each of the three axes) or vertical axis. This common method for categorizing PA intensities has been extensively used for the data processing of hip-mounted accelerometers in previous studies [27,28,29,30,31,32,33,34,35,36,37]. It is important to keep in mind that cut points are the thresholds of activity counts used to categorize activity as sedentary, light PA, moderate PA, vigorous PA, or very vigorous PA, and thus, is arbitrary in nature (see Figure 1). In detail, the classification of PA intensity is correlated with metabolic equivalents (METs—the amount of oxygen consumed when at rest) by using indirect calorimetry as reference standard [38]. Additionally, all previously established intensity thresholds have been determined by the activities chosen while conducting the validation studies. While adopting certain cut points to analyze accelerometer data in a specific study, researchers should follow the same data collection and data processing criteria that were used in the original validation study, making it challenging to apply the most appropriate intensity thresholds in free-living settings. As of the present, there are a number of waist-based cut points available for researchers. Some researchers have attempted to employ the waist-based cut points in the processing of wrist-derived accelerometer data (e.g., ActiGraph Link GT9X). Indeed, the ActiGraph company has suggested that users “uncheck” the box within its companion software (ActiLife, ActiGraph, Pensacola, FL, USA) for wrist-worn data collection if using cut-off points validated for waist-worn accelerometers. The idea of this checkbox is to classify the wrist-worn data to different PA intensities based on cut-off points validated for waist-worn accelerometers. However, this approach unrealistically overestimates individuals’ PA, particularly vigorous intensity PA, in free-living settings.

Recently, researchers have established wrist-worn cut points for different brands of devices. For instance, Johansson et al. [24] developed wrist-worn cut points for young preschoolers (15–36 months). For children, Crouter et al. [7] developed a regression model and cut points for the dominant wrist and recommended using the regression model only. Chandler et al. [39] and Trost et al. [40] developed different cut points for the non-dominant wrist. Similarly, Staudenmayer et al. [41] and Hildebrand et al. [42] developed cut points for the dominant and the non-dominant wrists for adults, respectively. Yet, the accuracy and reliability of these cut points need to be further confirmed by empirical studies [43]. For example, the authors used the cut points [7,42] for the pediatric population data in one of their studies. They found that vigorous intensity PA outputs looked unrealistically high (i.e., more than 3 h per day) with almost zero moderate intensity PA. In addition, researchers indicated that cut points validated for the hip-placed accelerometer could not accurately classify sedentary behavior while being applied to wrist-worn accelerometer data. Hence, they called for the need for the derivation and validation of wrist-worn cut points [44].

#### 3.1.2. Step-Based Cut Points

To avoid inaccurate output from cut points, researchers have employed the minute-by-minute stepping rate analysis to sort raw wrist accelerometer data and determine sedentary behavior and PA intensities. Indeed, this methodology has been employed to determine sedentary behavior, light physical activity (LPA), and moderate-to-vigorous PA (MVPA) in a number of studies conducted in free-living settings [45,46]. Specifically, researchers have used the following cut points to calculate steps per minute: sedentary behavior: 0–19; LPA: 20–99; MVPA: ≥100. A 60 s epoch was used to facilitate the assessment of participants’ minute-by-minute stepping rate [47]. To obtain valid data other than steps per day, participants’ raw data were first processed by manufacturer’s software and then exported to and analyzed in Microsoft Excel (Microsoft Inc.; Redmond, Washington, DC, USA) where “COUNTIFS” and “AVERAGE” functions were utilized to determine sedentary behavior, LPA, and MVPA time.

It should be noted that the minute-by-minute step threshold-based approach only considers one’s steps during walking. Yet, many human movement activities include complex bodily movements that may engage the upper body and go beyond simple ambulation. That is, one’s upper body movements will not be captured well by the simple rendering of steps taken. Therefore, many body movements beyond walking cannot be captured, meaning that an individuals’ PA might be underestimated when using this approach. In fact, the wrist-worn accelerometer detected more steps (2500 steps) than the hip-worn accelerometers in free-living settings [23].

#### 3.1.3. Gravitational Unit-Based Cut Points

There are several brands of accelerometers which provide raw accelerometer data, including GENEActiv, ActiGraph, and Axivity. The raw data can be retrieved from both wrist- and hip-worn accelerometers; therefore, researchers will be able to analyze the wrist-worn accelerometer data through appropriate approaches, such as GGIR cut points, which have been increasingly employed in the scientific communities.

Recently, researchers pre-processed the raw accelerometer signal, and then applied cut points to classify the intensity of PA, based upon the Euclidean norm minus one (ENMO) gravitational unit. GGIR is such a tool to implement the gravitational unit-based threshold approach. GGIR is an open-source R-package (http://cran.r-project.org, accessed on 10 August 2021) used to process multi-day raw accelerometer data for analyzing physical behaviors [48,49]. In detail, the raw data refer to the gravitational acceleration (gravity or milligravity (mg)), as this is used for the calibration of movement and gravitational acceleration. Notably, previous generations of accelerometers usually stored the data with various formats, which makes the outputs restricted by specific accelerometer brands. Thanks to the evolution of technology, most of the currently available triaxial accelerometers are able to store the raw data, which allows GGIR to process and analyze the raw data and generate meaningful physical behavior variables for statistical analyses. The GGIR outputs include each day and average estimates of PA, physical inactivity, and sleep. Simply stated, GGIR is considered a great tool for researchers processing raw accelerometer data and extracting insightful PA and sleep variables and comparing the findings from different accelerometers.

**Figure 2:**
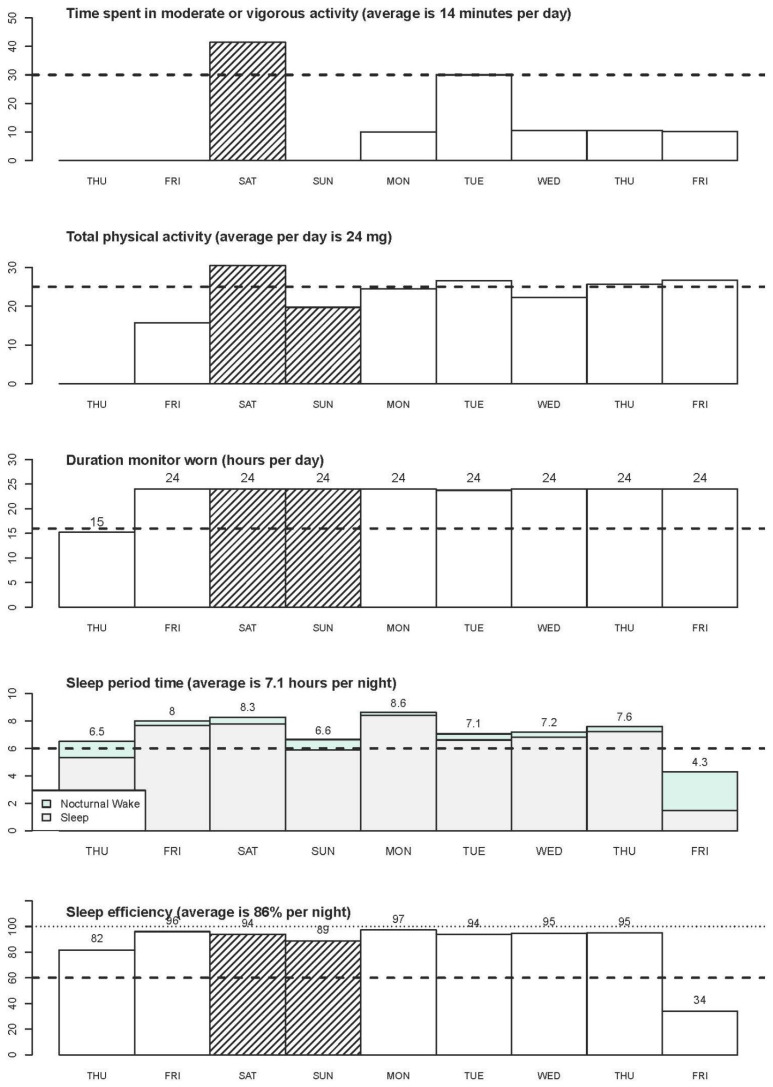
GGIR Visual outputs of PA, wearing time and sleep variables.

**Figure 3:**
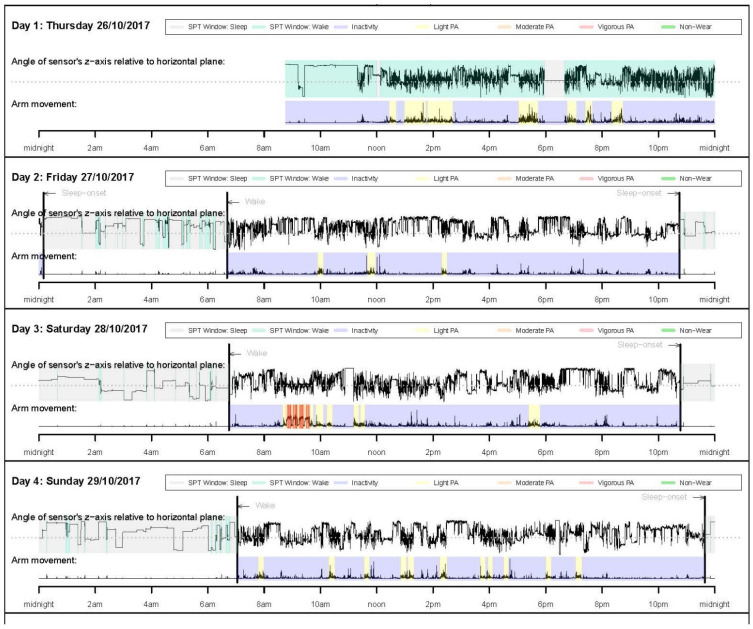
Daily PA and sleep patterns.

The GGIR R-package and a step-by-step simple example of using GGIR can be found in the literature [10]. The use of GGIR allows researchers to examine and compare studies that used accelerometers from different manufacturers. The sample summary outputs from GGIR provide information on key physical behavior variables, such as average daily wear time, time spent in moderate and/or vigorous PA, the amount of PA, sleep duration, and sleep efficiency (see Figure 2 and Figure 3). In addition, the summary report also provides visual patterns of PA and sleep patterns for each individual wearing day.

The advantages and disadvantages of using GGIR are listed as follows. Advantages include the following: (1) the GGIR R-package includes a broad set of functionalities that allow researchers to process raw data for 24 h/day, 7 days/week, based on different needs and analyzing free-living physical behaviors utilizing evidence-based methods; (2) GGIR can process raw accelerometer data recorded from different accelerometer brands (e.g., ActiGraph, GENEActiv, Axivity) and specifies the input arguments and the selection of output variables; (3) as a software for processing raw accelerometer data, GGIR encourages researchers to use raw data to fully understand the importance of PA and sleep for health; (4) as an open-source software, GGIR can be adapted and extended, based on different research needs, and the package team is continually developing more functions based on feedback from users around the globe. On the other hand, the disadvantages of using GGIR include the following: (1) GGIR can detect inactivity time rather than sedentary time (i.e., time spent sitting or reclining with low energy expenditure during waking time). However, GGIR defines physical inactivity not only in sitting or reclining position, but also includes standing without moving. Thus, caution is needed when interpreting the GGIR-generated inactivity summary, which is different from sedentary behavior; (2) in order to analyze full-day activities and sleep, GGIR requires at least 16 h of wear time to be considered valid. As GGIR has been developed for processing multi-day PA and sleep, this approach may not be suitable for studies measuring activities less than 16 h; (3) MVPA is calculated based on a 5 s epoch and this may sometimes overestimate individual’s actual MVPA; (4) running GGIR may require an intermediate level of running R and R studio software, in that it requires researchers to understand the codes for generating different metrics. Moreover, GGIR outputs include over 100 outcomes and thus, researchers need to fully understand the variables and select appropriate outcomes for interpretation.

### 3.2. Machine Learning Approaches

Machine learning is a subset of artificial intelligence, which has gained much attention over the past two years. Machine learning involves the creation of algorithms that can modify themselves without the need for human intervention to generate desired output(s) by feeding itself through a network of structured data. It uses algorithms to deconstruct the raw data, learn from that data, and make knowledgeable decisions, based on what it has learned. Deep learning, a subcomponent of machine learning, generates algorithms that have several layers, each one providing a distinctive interpretation of the data, which it feeds on. A network of such algorithms is called an artificial neural network, given its attempt at emulating the function of the human neural networks within the brain. Deep learning constructs algorithms in multiple layers to create an artificial neural network that is capable of learning and making intellectual decisions on its own. Thus, deep learning is what drives the most human-like artificial intelligence [50].

The latest innovations in deep learning algorithms and technologies have provided novel and effective paradigms, by which, one can obtain comprehensive learning models from big, complex data. Deep learning algorithms are capable of learning data representations with multiple levels of abstraction through computational models, based on multi-layered neural networks [51]. Furthermore, this general-purpose methodology of artificial intelligence is capable of learning data-based relationships, without having to define the data a priori [52]. Indeed, this capability to develop predictive models without the need for strong underlying assumptions of the mechanisms—which are typically unknown or insufficiently defined—is a major inherent benefit of utilizing deep learning approaches [53]. In detail, a normal deep learning workflow includes four steps, as follows: (1) harmonization of the data; (2) learning of representation data; (3) fitting of the model; (4) evaluation of the model [53]. Each layer of a deep learning system yields a data-based representation of the observed patterns as it receives inputs from the below layer(s) by augmenting a local, unsupervised criterion [54,55]. That is, deep learning algorithms disregard the need for human engineers and, rather, learn from the provided data using the preceding general-purpose learning procedures [51,54,55]. With recent breakthroughs in unsupervised pre-training [56], novel methods of preventing over-fitting [57], and with the employment of general-purpose processing to expedite computations of complex data, deep learning models have enabled effective, reliable, and fast solutions for many practical tasks. For instance, deep learning algorithms have demonstrated efficacy for identifying and/or discovering intricate structures in complex, multi-dimensional data and have achieved high success for the detection of objects in images [58], the recognition of speech [59], and the processing and translation of natural language [60,61]. With these advances, deep learning approaches have been examined and utilized for their effectiveness for translating complex biomedical data into improved healthcare and human health [54].

Within the past decade, deep learning approaches have been applied to streamline and improve different facets of the healthcare system. For instance, deep learning algorithms have been employed to improve clinical imaging data and have demonstrated effectiveness for the accurate classification of skin cancer [62], utilizing brain MRIs for the early diagnosis of Alzheimer’s disease [63], and for predicting osteoarthritis risk through the automatic segmentation of MRIs of knee cartilage [64]. Furthermore, the application of deep learning architecture has been used to enhance electronic health records and has shown efficacy for using patients’ clinical status to predict the incidence of future disease(s) [65]—using longitudinal electronic health records to predict chronic obstructive pulmonary disease and congestive heart failure [65]—and for using pediatric intensive care unit patients’ clinical measures to more accurately classify their diagnoses [66]. Moreover, the study of genomics has been enhanced by deep learning and has facilitated the use of protein sequences to predict protein backbones [67], using gene expression profiles to better classify cancers [68], and using DNA sequences to better predict chromatin marks [69]. Lastly, deep learning has helped improve mobile healthcare technologies and has demonstrated effectiveness for the analysis of local field potentials signals and electroencephalograms [70], detecting Parkinson’s disease patients’ freezing of gait [71], and for improved health monitoring though the identification of photoplethysmography signals [72]. Based on the preceding advances in traditional medicine with the utility of deep learning, the field of PA and health promotion has recently followed suit and has started to utilize powerful deep learning approaches to more accurately characterize and analyze free-living human activity and sleep patterns. Specifically, novel deep learning approaches have been applied to accelerometry—the preferred device-based measure of human activity in free-living settings—for its accuracy and acceptability by participants [73,74,75].

Historically, one major issue with traditional methods of analyzing accelerometer-derived PA data was that manufacturers’ proprietary (i.e., undisclosed) algorithms reduced the raw data to “counts”, which are dimensionless units [76]. From there, PA levels (e.g., SB, LPA, MVPA) are defined using the previously discussed “cut points” [75] and the duration spent in each pre-defined intensity category is then calculated. Thus, although commonly referred to as an objective PA measure, decisions made by the researchers handling these data (e.g., selected cut points and valid non-wear/day criteria) are subjective and differ between studies and devices used, thereby producing inconsistent PA estimates [76,77]. Because of this, researchers have advocated for transparency and have requested and successfully gained access to manufacturers’ raw (i.e., unfiltered) PA data outputs, which are large and often difficult to manage, analyze, and interpret [78]. Indeed, compared with traditional PA counts derived from wrist-worn accelerometers, raw data metrics (e.g., ENMO) have demonstrated promise for improving between-device comparability [79]—another common issue in device-based PA research. That said, researchers in this field of inquiry have evaluated the effectiveness of deep learning algorithms and have demonstrated promising results for predicting various PA types and/or intensities as well as sleep/wake identification [80,81,82].

As is known, wrist-worn accelerometers are associated with greater compliance. However, validated algorithms for predicting activity type from wrist-worn accelerometer data are lacking. Trost et al. [83] used a machine learning algorithm to compare PA recognition rates of both hip- and wrist-appended accelerometers and found that algorithms associated with both wear locations achieved good accuracy for the classification of PA intensities, which has allowed researchers to more confidently use either placement for recognition of PA. Similarly, Mathur et al. [84] successfully performed deep data augmentation training to address heterogeneities in wearable sensing devices’ software and hardware. Furthermore, Jiang et al. [85] validated a deep learning model to accurately impute missing PA data—a common issue with free-living, device-determined PA data—and observed it to outperform other known methods of imputing missing activity data. Furthermore, van Kuppevelt et al. [86] employed an unsupervised classification approach, which successfully learned different human PA behaviors, based on the accelerometer-derived data it observed. In detail, this machine learning approach demonstrated the ability to pool multiple PA data metrics without the need for costly calibration studies, thereby offering a cost-effective, multi-dimensional description of human PA behaviors. Lastly, Sathyanarayana et al. [87] employed a convolutional neural network on the raw data output from wrist-based accelerometer and observed it to eliminate the need for data pre-processing and to simplify the overall workflow to analyze ActiGraph data for PA and sleep research.

Deep learning frameworks have also been utilized for sleep–wake detection with the use of accelerometer-derived acceleration and heart rate variability data [88]. Taking into consideration the high sampling rate of movement acceleration data and its temporal dependency, Chen et al. examined a deep learning approach (local feature-based long short-term memory (LFLSTM)) which was successfully able to learn high-level features and accurately detected and classified human sleep–wake behaviors—findings echoed by Li et al. [89]. Similarly, Hafezi et al. [90] used deep learning to accurately estimate the severity of adults’ sleep apnea and validated this approach against full night polysomnography—the gold standard for sleep–wake detection. Moreover, Cho et al. [91] employed end-to-end deep learning architecture (Deep-ACTINet) to analyze wrist ActiGraph data for the automatic detection of the human sleep–wake cycle. This approach did not require a feature engineering method and only used noise-canceled raw activity signals recorded during sleep. This study demonstrated Deep-ACTINet to extract the accelerometer data more effectively and efficiently than traditional feature engineering (machine learning) methods and to yield more accurate separation of sleep and wake behaviors. Likewise, Sathyanarayana et al. [92] used deep learning for the correction of sleep quality prediction from wrist ActiGraph data.

**Figure 4:**
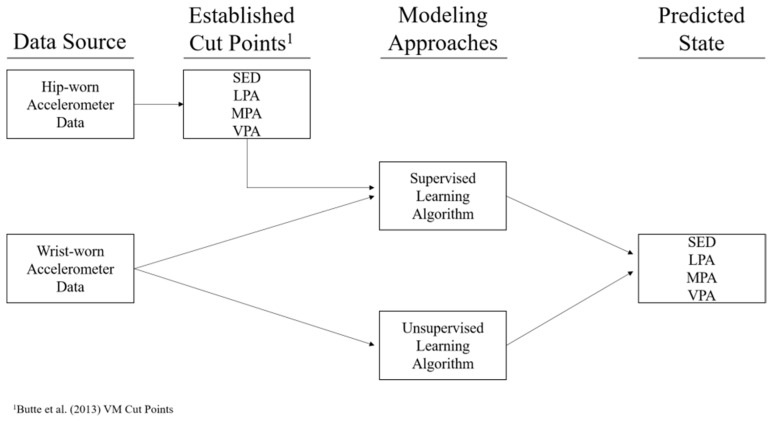
Modeling process diagram ([94]; permission from Li et al., 2020). Note: SED—sedentary; LPA—light physical activity; MPA—moderate physical activity; VPA—vigorous physical activity.

Lastly, Trost et al. [93] adopted machine learning approaches to develop wrist-based prediction models for activity type and intensity in preschool children and compared them to the activity counts-based/cut point approach. They found that machine learning algorithms were useful in predicting physical behaviors in this population and that the combination of wrist and hip accelerometer data worked better. Li et al. [94], using machine learning approaches (See Figure 4), developed PA intensity cut points for wrist-worn accelerometers to assess preschoolers’ PA (i.e., sedentary, light PA, moderate PA, vigorous PA, and MVPA) in comparison to a previously established wrist-worn reference [95] and to an established hip-worn reference [96]. They found that machine learning approaches have potential for establishing cut points for wrist-worn accelerometers and to accurately classify PA levels in this population. Chikhaoui et al. [97] also employed a deep learning algorithm (stacked auto-encoder matrix factorization) to automatically learn suitable features and for dimensionality reduction. This approach achieved superior performance compared with all other benchmark approaches for sleep–wake detection (e.g., full night polysomnography). Although machine learning has been used in this field for over a decade, it is gaining traction due to its increased accessibility [98]. However, although deep learning algorithms have demonstrated good accuracy for predicting various components of PA, their application in free-living settings currently needs further research [99].

Machine learning involves a wide range of techniques to mechanize and automate the learning of non-linear and/or high-dimensional data patterns with predictive ability (supervised learning) or reduction in data (unsupervised learning) as its fundamental priority [81]. While machine/deep learning methods are in their infancy, their utility for diagnostic/prognostic purposes and PA behavior change settings are worth implementing. Advantages of machine learning approaches include the following: (1) machine learning’s utility for the generation of data-driven hypotheses; (2) the potential of model training in one dataset and the updating of this model in another dataset; (3) machine learning’s ability to handle multi-dimensional data; (4) machine learning’s capacity to locate non-linear patterns. Disadvantages are as follows: (1) estimates of time spent in different intensities of PA, sedentary behavior, or types of PA (e.g., cycling, running, walking) can be optionally articulated in unbouted and/or bouted behavior with machine/deep learning approaches; (2) it can be problematic to interpret (especially among researchers with no background in computer science) how the end results were obtained and, ultimately, their implication for sedentary behavior and PA guidelines; (3) machine learning approaches have also been observed as “data hungry” and researchers outside of the field of computer science may shy away from employing these methods as they are generally perceived as “computationally demanding”; (4) sensitivity and overfitting to biases that are potentially unknown to researchers in the training data are risks.

## 4. Opportunities and Challenges

The switch from hip-placed to wrist-worn accelerometers has signaled the focus on developing novel approaches to process raw acceleration data without overly relying on traditional cut points. Indeed, wrist-worn raw acceleration data have been increasingly utilized to capture individuals’ physical behavior patterns [48,49]. Several traditional methods and cut points have been developed to deal with raw wrist acceleration data [39,42], yet they appear to either overestimate or underestimate the outcomes [100]. Fortunately, some novel statistical and machine/deep learning approaches have been developed recently to process raw, wrist-derived acceleration data [41,101], offering unique advantages to process free-living physical behavior data. However, these approaches need to undergo the peer-review process. Thus, there is a need for accurate and appropriate methods to convert the wrist-worn accelerometer data into behavioral outcomes (e.g., time spent in MVPA, etc.) [102]. More systematic reviews in this area are also needed on a regular basis.

Using cut points, we will not be able to know the actual PA levels and prevalence of meeting the PA guidelines (or not) based on accelerometer data. There are less methodological challenges, but there is certainly a consensus challenge. An established standard for the measurement of physical behaviors does not exist due to the complexity of the behaviors. For example, PA is multifaceted and includes different domains (e.g., leisure, occupation, transport, household), dimensions (e.g., frequency, duration, intensity, type), correlates, and determinants (e.g., where, when, why, with whom). Therefore, the appropriateness of any measure of physical behaviors depends on study aims. That said, there is no “one size fits all” solution.

The advancement in accelerometry over the last decade comes with the development of companion software, such as ActiLife. However, manufacturers’ software use of proprietary algorithms, which are unavailable to the public, make it difficult to process the data to obtain activity counts. Even with ActiLife, more algorithms need to be developed for sleep data. For example, currently, only 2 algorithms with 60 s epochs are available for sleep data. With GGIR, only sleep duration and sleep efficiency can be retrieved as of today, and thus, researchers should develop more open-source resources in R to generate more sleep outcomes relevant to health. As the code is open source, it can be used in part or as a whole, making it flexible to fit a variety of research needs. It further facilitates a reproducible analysis of raw data, which is key to generating conclusions in clinical and observational research settings. This demonstrates the importance of developing open-source resources based on researchers’ needs. Additionally, there is a need to use more machine/deep learning strategies to process sedentary and sleep data.

In the past several years, more researchers have been advocating the use of 24 h movement behaviors. Some countries, including Canada, Australia, and South Korea, established guidelines for 24 h movement behaviors (e.g., sedentary behavior, PA, sleep). Hence, we recommend recording raw wrist data for complete days (i.e., 24 h periods) so that the collected data will have maximum potential for future analyses. In addition, epidemiological studies are the basis for establishing the PA guidelines. The questionnaires queried behaviors that often occur within specific contexts, such as one’s occupation, household tasks, sports and recreation activities, transportation, walking, or bicycling. Even though accelerometer-based devices accurately measure movement associated with PA, devices are not simply better measures of the same construct captured by questionnaires. Therefore, evaluation of PA guideline adherence based on accelerometer outcomes is insufficient, because the behavioral metrics used to develop the guidelines differ conceptually from device-based measures of MVPA.

Lastly, large-scale PA intervention studies using accelerometers are limited due to the relatively high cost of accelerometers in such research settings. Thus, the development of lower cost, yet high-quality accelerometers are needed to facilitate greater use of these devices in big surveillance studies and PA intervention studies. Research-grade accelerometers may also learn from the commercial, wearable-like smart watches, to not only provide instant feedback but to also offer companion apps and online social communities. For example, Davoudi et al. [103] examined the validity of Samsung Gear S smartwatch while using ActiGraph accelerometer as the criterion and found that this smartwatch was relatively accurate in assessing individual activity recognition, activity intensity detection, major body movement location detection, and locomotion detection tasks. Pope and colleagues [104,105] compared Apple Watch, Fitbit Surge HR, TomTom Multisport Cardio Watch, and Microsoft Band against ActiGraph accelerometer with two different samples. They observed that smartwatch average/peak heart rate measurements were moderately valid, yet smartwatch energy expenditure measurements were less valid [104]. Among them, Apple Watch and Microsoft Band had the most accurate EE estimation [105]. More research development is needed in this area.

## 5. Directions for Future Studies

- Combine the 24 h raw accelerometer data processing/analysis and survey contents via the app questionnaire approach to establish guidelines for physical behaviors.
- Translate the findings of accelerometer-driven physical behaviors to meaningful information for public health guidelines.
- Investigate the mutual effects/correlates among physical behaviors and relevant health outcomes by employing various raw accelerometer data processing and analytical approaches and then crosscheck the findings.
- Identify which raw accelerometer data processing and analytical approach is the most appropriate based on research purpose(s) and question(s).
- Explore the interactional effect of physical behaviors (PA, sedentary behavior, and sleep) and a variety of health outcomes, particularly the role of sleep, while also including other physical behaviors.
- Further examine the interactive effects of PA intensity and PA type, particularly light PA intensity, on individuals’ health outcomes with raw accelerometer data.
- Numerous commercially available wearable sensors (e.g., Fitbit, Apple Watch) use manufacturer-developed algorithms and do not make them open and transparent to the public, nor do they make raw data available. Yet, they all claim that their wearables are accurate and reliable. It is beyond the scope of this paper, but we recommend more studies aim to discern the validity and reliability of the accelerometers and commercial health wearables [106].

## 6. Conclusions

In summary, this paper comprehensively synthesizes different methods of analyzing physical behaviors from wrist accelerometer data. Although the findings from wrist-worn accelerometers should continue to be interpreted with caution, we recommend cutting-edge, yet appropriate analysis plans for wrist-worn accelerometer data in assessing physical behavior data. Specifically, GGIR and machine/deep learning hold great potential in processing raw, wrist-derived accelerometer data in the future. Further refinements of these methods and the development of other new methods to process wrist-worn accelerometer data are warranted to improve our understanding in this area of inquiry.

## Figures and Tables

**Table 1:** Similarities and differences of wrist- vs. hip-worn accelerometers.

	Similarities			Differences	
	Wrist-Worn	Hip-Worn		Wrist-Worn	Hip-Worn
**Epoch Length**	1 s, 5 s, 15 s, ≥60 s		**Wear time**	Daytime only (ranging from 10–16 h/day)	24 h/day
**Sampling Frequency**	30–100 Hz		**Device Placement**	Right hip (preferred) or left hip	non-dominant wrist or dominant wrist
**Filter**	Normal filter, low-frequency extension filters				

Note. The bold areas are the comparison categories.

## Data Availability

The references included in this study are available from the online database or on request from the corresponding author.
